# Enhancement of myogenic differentiation and myotube formation by stanozolol in porcine muscle satellite cells for cultured meat production

**DOI:** 10.5713/ab.260470

**Published:** 2026-07-27

**Authors:** Ji Hwan Ryoo, Keun Woo Kang, Hyun Su Jung, Su Min Park, Hyuk Cheol Kwon, Byeong Jun Choi, Eun Seo Min, Jeong Tae Do, Sung Gu Han

**Affiliations:** 1 Department of Food Science and Biotechnology of Animal Resources, College of Life Science, Konkuk University, Seoul, Korea; 2 Research Group of Food Processing, Korea Food Research Institute, Wanju, Korea; 3 School of Advanced Biotechnology, Konkuk University, Seoul, Korea

**Keywords:** Cell-cultured Meat, Muscle Satellite Cell, Myogenic Differentiation, Myostatin, Stanozolol

## Abstract

**Objective:**

Recent studies on cultured meat have shown that muscle satellite cells can be induced to form muscle-like tissues through proliferation and myogenic differentiation. Optimizing these processes is essential for reproducing the structure and texture of conventional meat; yet, liming myogenic differentiation remains a major technical challenge. Stanozolol (STZ), a synthetic anabolic steroid derived from testosterone, is known to enhance muscle protein synthesis and hypertrophy, but its effects on livestock-derived muscle cells for cultured meat production remain unclear. This study aimed to evaluate whether STZ enhances myogenic differentiation and myotube formation in primary porcine muscle satellite cells (PMSCs).

**Methods:**

PMSCs were treated with STZ at concentrations of 0.1, 0.5, and 1 μM. Cell viability and proliferation were assessed to determine the non-cytotoxic concentration range. Myogenic differentiation was evaluated by measuring the expression of myogenic differentiation 1 (MyoD), myogenin (MyoG), myosin heavy chain (MyHC), and myostatin (MSTN). Myotube formation was analysed by immunofluorescence staining, and the underlying mechanisms were investigated by examining phosphatidylinositol 3-kinase (PI3K)/serine/threonine kinase (AKT)/mammalian target of rapamycin (mTOR), Smad2/3, and forkhead box O3a (FoxO3a) signaling.

**Results:**

At concentrations ranging from 0.1 to 1 μM, STZ showed neither cytotoxicity nor significant PMSC proliferation. In differentiation media, STZ significantly increased the mRNA and protein expression of myogenic markers, including MyoD, MyoG, and MyHC, in a concentration-dependent manner. Notably, 1 μM STZ promoted the formation of bigger multinucleated myotubes. In addition, STZ decreased MSTN expression, activated PI3K/AKT/mTOR hypertrophic signaling, and suppressed Smad2/3 phosphorylation and FoxO3a activity.

**Conclusion:**

STZ promoted myogenic differentiation and myotube formation in PMSCs by enhancing myogenic marker expression and modulating hypertrophy- and atrophy-associated signaling pathways. These findings suggest that STZ may serve as an effective culture medium supplement for improving PMSC differentiation in cultured meat production.

## INTRODUCTION

Meat serves as a fundamental staple of the human diet and is a primary source of high-quality protein, providing a complete amino acid profile essential for physiological growth and maintenance [1]. However, conventional meat production is increasingly associated with substantial environmental pollution, animal welfare concerns, and antibiotic misuse. Furthermore, the persistent threat of infectious and zoonotic diseases continues to compromise the biosafety and long-term sustainability of livestock [2,3]. Recently, cellular agriculture has emerged globally to address growing concerns regarding environmental sustainability and food security [2,3]. Cultured meat offers a promising approach for producing edible muscle tissue from animal cells in a laboratory setting, effectively bypassing the environmental and ethical costs of conventional livestock farming [3]. The primary objective of cultured meat technology is to promote the differentiation of muscle satellite cells (MSCs) into mature muscle fibers, thereby producing a structured tissue that recapitulates conventional meat [4,5]. However, MSCs frequently experience a loss of myogenic potential during *ex vivo* expansion, and their capacity to form mature myotubes decreases over time [4]. Consequently, developing techniques to preserve and enhance MSC differentiation is a pivotal focus of current meat culture research [5].

Anabolic androgenic steroids (AASs) are a class of natural and synthetic hormones that are structurally similar to testosterone, and have been used clinically to treat conditions such as hypogonadism, osteoporosis, and aplastic anemia. Additionally, AASs have also been used to increase muscle mass and strength [6,7]. Physiologically, AASs act as potent anabolic agents that increase muscle protein synthesis, support myotube maturation, regulate the expression of key genes involved in muscle development, and promote myogenic differentiation. Additionally, AASs enhance the fusion of satellite cells into developing myotubes by binding to the androgen receptor (AR). Previous studies have demonstrated that AASs promote protein synthesis by activating the insulin-like growth factor 1 (IGF-1)/phosphatidylinositol 3-kinase (PI3K)/serine/threonine kinase (AKT)/mammalian target of rapamycin (mTOR) signaling pathway in an AR-dependent manner [7,8]. AASs enhance myotube formation by upregulating expression of key myogenic regulatory factors (MRFs), including myogenic differentiation 1 (MyoD) and myogenin (MyoG), thereby promoting myogenic differentiation of satellite cells [8,9].

Stanozolol (STZ) is a member of the AAS family and a 17α-alkylated testosterone derivative with potent anabolic and androgenic properties. In addition to its clinical applications in treating muscle-wasting conditions, STZ has been widely recognized as an ergogenic aid because of its ability to stimulate protein synthesis and hypertrophy in skeletal muscles [7,10]. Previous studies have reported that STZ reduces mitochondrial reactive oxygen species generation and attenuates acute exercise–induced oxidative stress in rat skeletal muscle [11]. In addition, STZ administration increases the bulk and diameter of type I muscle fibers in humans, indicating a hypertrophic response [12]. These findings suggest that STZ promotes muscle protein accretion and modulates muscle remodeling to support enhanced hypertrophic growth. Consequently, STZ is a promising culture medium supplement for optimizing the myogenic differentiation of MSCs in cultured meat production. However, studies on STZ have primarily been restricted to human tissue-based metabolism assays, veterinary studies, and rodent models [11,13]. Previous research has largely focused on STZ-induced injury and apoptosis in differentiated skeletal muscle cells rather than investigating its potential effects on the myogenic differentiation of MSCs [13]. Therefore, the objective of this study was to evaluate the efficacy of STZ as a supplement for enhancing myotube formation and optimizing cultured meat production. We specifically sought to determine whether STZ promotes the myogenic differentiation of primary porcine muscle satellite cells (PMSCs) and elucidate the underlying signaling mechanisms.

## MATERIALS AND METHODS

### Chemical and reagents

STZ was purchased from the Tokyo Chemical Industry. Dulbecco’s modified Eagle’s medium/F-12 nutrient mixture (DMEM/F-12), DMEM/High Glucose, fetal bovine serum (FBS), penicillin–streptomycin (PS), trypsin-ethylenediaminetetraacetic acid (EDTA), and insulin (INS) were purchased from WELGENE. Horse serum (HS), penicillin–streptomycin–glutamine (PSG), and phosphate-buffered saline (PBS) were obtained from Gibco. Recombinant human basic fibroblast growth factor (bFGF) was purchased from Bio-Techne. Cell culture plates (96-, 24-, and 6-well) were purchased from SPL Life Sciences.

### Isolation of porcine muscle satellite cells

PMSCs were isolated from the skeletal muscle tissues from the hind leg femurs of 1-day-old piglets, as previously described [14]. The collected tissues were washed three times with PBS (Gibco) containing 10% PS (Gibco). Two grams of tissue per sample was minced and digested in DMEM/F-12 containing 2 mg/mL collagenase D (Roche), 1 U/mL Dispase II (Roche), 0.25% trypsin-EDTA, and 10% PS. The mixture was incubated at 37°C for 1 h with gentle agitation. After enzymatic digestion, the homogenate was sequentially filtered through 100 μm and 70 μm cell strainers and neutralized with DMEM/F-12 supplemented with 15% FBS. The filtrate was centrifuged at 250×g for 5 min, and the supernatant was treated with ammonium–chloride–potassium lysing buffer (Gibco) on ice for 5 min to remove erythrocytes. The resulting cell pellet was resuspended in a proliferation medium composed of DMEM/F-12 supplemented with 15% FBS, 1% PS, and 10 ng/mL bFGF. The cell suspension was seeded onto 0.1% gelatin-coated culture plates and incubated at 37°C for 1 h. Subsequently, unattached cells were collected and transferred to new gelatin-coated plates for further culture.

### Cell culture and treatments

PMSCs (passages 3–4) were used for all experiments and cultured in a humidified incubator at 37°C under 5% CO_2_. The cells were maintained in growth medium (DMEM/F-12) supplemented with 15% FBS, 1% PSG, and 10 ng/mL bFGF, as previously described [15]. The growth medium was replaced daily, and the cells were subcultured to approximately 80% confluence using 0.05% trypsin-EDTA. Once the PMSCs reached 100% confluency, the growth medium was replaced with differentiation medium (DMEM/High Glucose supplemented with 5% HS, 1% PSG, and 1 μg/mL INS) containing STZ at concentrations of 0.1, 0.5, and 1 μM. The cells were differentiated for 1 or 7 days, and the differentiation medium was refreshed daily. STZ was dissolved in dimethyl sulfoxide (DMSO).

### Cell viability

PMSCs were seeded at a density of 2×10^4^ cells/well in 96-well plates and treated with STZ (0.1, 0.5, 1, 5, 10, and 20 μM) for 24 h. Following treatment, the medium was replaced with 110 μL of fresh medium containing 10 μL of 3-(4,5-dimethylthiazol-2-yl)-2,5-diphenyltetrazolium bromide (MTT) solution (5 mg/mL in PBS) and incubated at 37°C for 3 h. Then, 90 μL of medium was removed and replaced with 180 μL of acidic isopropanol to solubilize the insoluble formazan crystals. Thereafter, the plates were incubated at 37°C for 1 h. Optical density (OD) was measured at 570 and 630 nm using an Epoch spectrophotometer (BioTek Instruments). Cell viability was calculated by subtracting the OD at 630 nm from that at 570 nm using the following formula:


(1) $$\text{Cell viability }(\%)=({\text{OD}}_{\text{experimental sample}}/{\text{OD}}_{\text{control sample}})\times 100\;(\%).$$

### Cell proliferation analysis

PMSCs were seeded at a density of 2.5×10^5^ cells/well in 6-well plates and treated with various concentrations of STZ (0.1, 0.5, and 1 μM) for 1, 3, and 5 days. Cells attached to the bottom of the wells were detached using trypsin-EDTA and counted using an optical microscope and a hemocytometer (Hausser Scientific). Before cell counting, cell morphology was imaged and captured using Nikon Eclipse Ti2-U and Nikon Eclipse Ts2R cameras (Nikon).

Cell proliferation was analyzed using a D-Plus cholecystokinin (CCK) kit (Dongin Biotech). PMSCs were seeded at a density of 1×10^4^ cells/well in 96-well plates and treated with STZ (0.1, 0.5, 1, 5, 10, and 20 μM) for 48 h. Following treatment, the medium was replaced with 110 μL of fresh medium containing 10 μL of CCK solution and incubated at 37°C for 3 h. OD was measured at 450 nm using an Epoch spectrophotometer. Cell proliferation was calculated using the following formula:


(2) $$\text{Cell proliferation }(\%)=({\text{OD}}_{\text{experimental sample}}/{\text{OD}}_{\text{control sample}})\times 100\;(\%).$$

### RNA extraction and real-time polymerase chain reaction

MyoD, MyoG, myostatin (MSTN), and myosin heavy chain (MyHC) mRNA levels were measured using real-time polymerase chain reaction (RT-qPCR). PMSCs were seeded at a density of 2×10^5^ cells/well in 6-well plates coated with 0.15% gelatin and incubated for 1 and 7 days in a differentiation medium supplemented with STZ (0.1, 0.5, and 1 μM). RNA was extracted using TRIzol reagent (Thermo Fisher Scientific), and cDNA was synthesized using the TOPscript RT DryMIX kit (Enzynomics). Gene expression was evaluated using the RT-qPCR Smart mix (BIOFACT) and the RT-qPCR system (Roche LightCycler 96 System). The RT-qPCR reaction was performed under thermal cycling conditions consisting of 60 cycles of denaturation at 95°C for 10 s, annealing at 60°C for 10 s, and extension at 72°C for 10 s. The mRNA expression levels were quantified using the 2−ΔΔCt method, with normalization performed using glyceraldehyde 3-phosphate dehydrogenase (GAPDH) as an internal control. The primer sequences used for RT-qPCR were: MyoD, (F) 5′-CGT GCA AAC GCA AGA CCA CTA A-3′, (R) 5′-CGC TGA TTC GGG TTG CTA GAC-3′; MyoG, (F) 5′-ATC ATC TGC TCA CAG CTG ACC CTA-3′, (R) 5′-TCA GTT GGG CAT GGT TTC ATC TGG-3′; MSTN, (F) 5′-CAG TAA ACT TCG CCT GGA AAC AGC-3′, (R) 5′-TCA TCT TCC AAG GAG CCA TCA CTG-3′; MyHC, (F) 5′-AGG AAG AGT ACG CCA AGG GGA AAA-3′, (R) 5′-GAG GCC CGA GTA GGT ATA GAT CAT-3′; GAPDH, (F) 5′-TGA AGG TCG GAG TGA ACG GAT TTG-3′, (R) 5′-CAA CAT ACG TAG CAC CAG CAT CAC-3′.

### Protein extraction and western blot

PMSCs were seeded at a density of 2×10^5^ cells/well in 6-well plates coated with 0.15% gelatin and incubated for 1 and 7 days in a differentiation medium supplemented with STZ (0.1, 0.5, and 1 μM). The cells were harvested and lysed on ice using a radioimmunoprecipitation assay buffer (Elpis Biotech) supplemented with a protease inhibitor cocktail (Abbkine). The lysates were collected in a 1.7 mL tube and centrifuged at 17,000×g at 4°C for 20 min. Protein concentrations in the supernatants were quantified using a bicinchoninic acid protein assay kit (Thermo Fisher Scientific). Equal amounts of protein samples were separated by performing sodium dodecyl sulfate-polyacrylamide gel electrophoresis and then transferred onto 0.2 μm pore-size nitrocellulose membranes (GE Healthcare Biosciences) using a semi-dry electrophoretic transfer cell (Bio-Rad Laboratories). The membranes were blocked with 5% bovine serum albumin for 1 h 30 min at 25°C, followed by overnight incubation at 4°C with primary antibodies: anti-MyoD (1:3,000; Cell Signaling Technology), anti-MyoG (1:3,000; Thermo Fisher Scientific), anti-phospho-PI3K (1:2,000; ABclonal), anti-PI3K (1:2,000; ABclonal), anti-phospho-AKT (1:1,000; Cell Signaling Technology), anti-AKT (1:1,000; Cell Signaling Technology), anti-phospho-mTOR (1:1,000; ABclonal), anti-mTOR (1:1,000; ABclonal), anti-phospho-Smad2/3 (1:1,000; Cell Signaling Technology), anti-Smad2/3 (1:1,000; Santa Cruz Biotechnology), and anti-GAPDH (1:5,000; Cell Signaling Technology). The membranes were washed three times with Tris-buffered saline containing Tween 20 buffer for 15 min and then incubated with goat anti-rabbit IgG conjugated with horseradish peroxidase (1:7,500; Enzo Life Sciences) and goat anti-mouse IgG H&L conjugated with horseradish peroxidase (1:7,500; Abcam) for 1 h 30 min at 25°C. Protein bands were visualized using an enhanced chemiluminescence detection reagent (32209; Thermo Fisher Scientific; DG-WP500 and DG-WF100; DoGenBio). GAPDH was used as the internal control for normalization. The density of each protein band was quantified using ImageJ software (ver. 1.52a; National Institutes of Health).

### Immunofluorescence

PMSCs were seeded at 5×10^4^ cells/well in 24-well plates coated with 0.15% gelatin and incubated for 1 h, 24 h and 7 days in a differentiation medium supplemented with STZ (0.1, 0.5, and 1 μM). The PMSCs were fixed with 4% paraformaldehyde for 15 min and permeabilized with 0.1% Triton X-100 for 10 min. After blocking buffer with 3% bovine serum albumin and 2% donkey serum (GTX2320040-01; GeneTex) for 1 h 30 min at 25°C, the cells were incubated with primary antibodies: anti-MyHC (1:200; Thermo Fisher Scientific) and anti-forkhead box O3 (FoxO3a, 1:200; Sigma-Aldrich) for 24 h at 4°C. After washing with 0.1% Tween 20, the cells were incubated with DyLight 488-conjugated donkey anti-rabbit IgG H&L (1:1,000; Bethyl Laboratories) and donkey anti-rabbit IgG H&L Alexa Fluor 647 (1:1,000; Abcam) for 1 h 30 min at 25°C. After fixing with 4% paraformaldehyde for 10 min, cell nuclei were stained with 4′,6-diamidino-2-phenylindole dihydrochloride (DAPI; 1 μg/mL) for 15 min and washed three times with 0.1% Tween 20. Immunofluorescence-stained PMSCs were imaged using Nikon Eclipse Ti2-U and Nikon Eclipse Ts2R cameras. Myotube formation was quantified using ImageJ software. All fluorescence signals were quantified using identical threshold settings. The MyHC-positive area was expressed as a percentage of the total field area. The fusion index was calculated as the percentage of nuclei incorporated into the multinucleated myotubes relative to the total number of nuclei.


(3) MyHC-postive area (%)=(MyHC-stained area/Total area)×100

(4) Fusion index (%)=(Nuclei in myotubes/ Total nuclei)×100.

### Statistical analysis

All experiments were conducted at least three times and are presented as the mean±standard error. Statistical significance was analyzed using the Dunnett’s t-test. Data were analyzed using SPSS-PASW Statistics (ver. 22.0; SPSS). Statistical significance was set at p<0.05.

## RESULTS

### Effects of stanozolol on cytotoxicity and proliferation of porcine muscle satellite cells

The MTT and CCK assays were performed to determine the optimal STZ concentration in the culture medium. The effects of STZ were evaluated across a concentration range of 0.1–20 μM at 24 and 48 h. The control and vehicle control (Veh; 0.2% DMSO in the growth medium) groups showed no significant differences. The viability and proliferation of PMSCs were not significantly affected by STZ at concentrations ranging from 0.1 to 1 μM. However, STZ significantly reduced cell viability at 5–20 μM in the MTT assay and at 20 μM in the CCK assay, compared with those of the control group (p<0.05) (Figures 1A, 1B). Accordingly, concentrations of 0.1, 0.5, and 1 μM STZ were selected for subsequent experiments. Although 1 μM STZ increased the total cell number on day 1 (p<0.05) (Figure 1C), no significant differences were observed among the groups on days 3 and 5 (Figure 1C). Morphological observations showed no apparent changes in cell morphology across the treatment groups (Figure 1D). These findings demonstrated that 0.1–1 μM STZ did not induce cytotoxicity or changes in cellular morphology.

### Effects of stanozolol on terminal differentiation and myotube formation in porcine muscle satellite cells

Myotube formation was observed in PMSCs cultured in the differentiation medium for 7 days, and myotube size increased in an STZ concentration-dependent manner. In addition, MyHC fluorescence intensity and multinucleated myotube formation increased markedly as the STZ concentration increased (Figure 2A). Both the MyHC-positive area and fusion index increased significantly in a STZ concentration-dependent manner, reaching 35.3% and 59.5%, respectively, at 1 μM STZ (p<0.001) (Figure 2B). The fusion index reflects the capacity for myogenic differentiation by measuring the proportion of fused myoblasts. Our data showed that the fusion index of MyHC was significantly higher in 0.1, 0.5 and 1 μM STZ than in that of the control (p<0.001) (Figure 2C). These results indicated that STZ promoted myogenic maturation in a concentration-dependent manner.

### Effects of stanozolol on mRNA and protein expression of myogenic markers in porcine muscle satellite cells

In PMSCs treated with 0.5 and 1 μM STZ, mRNA levels of MyoD, MyoG, and MyHC were significantly higher than that of the control group on day 1 (p<0.05) (Figure 3A). On day 7, mRNA expression of these genes was significantly higher in PMSCs treated with 0.1, 0.5, and 1 μM STZ compared with that of the controls (Figure 3B). Consistently, MyoD and MyoG protein expression were significantly higher in the 0.1, 0.5 and 1 μM STZ treatment than in the control group on days 1 and 7 (p<0.05) (Figure 3C). In contrast, MSTN mRNA levels were significantly decreased in the 1 μM STZ on day 1 and in the 0.1–1 μM STZ on day 7 compared with that of the control group (p<0.05) (Figures 3A, 3B). These results suggest that STZ promoted myogenic differentiation of PMSCs by upregulating expression of key myogenic markers (MyoD, MyoG, and MyHC) and downregulating that of MSTN. Based on these findings, 1 μM STZ was selected as the most effective concentration for inducing myogenic differentiation. Accordingly, subsequent signaling pathway analyses were conducted by comparing the control and 1 μM STZ-treated groups.

### Effects of stanozolol on phosphatidylinositol 3-kinase/serine/threonine kinase/mammalian target of rapamycin pathway in porcine muscle satellite cells

The phosphorylation levels of PI3K, AKT, and mTOR were increased in PMSCs treated with 1 μM STZ compared with those of the control group at 1 and 24 h (Figure 4). PI3K phosphorylation was significantly higher than that in the control group at 24 h (p<0.05) (Figure 4A). In addition, phosphorylation of AKT and mTOR was significantly increased as early as 1 h after 1 μM STZ treatment and remained elevated up to 24 h (p<0.05) (Figures 4B, 4C). These results demonstrated that STZ induced sustained activation of the PI3K/AKT/mTOR signaling pathway in PMSCs for up to 24 h, suggesting that the promyogenic effects of STZ are associated with hypertrophy-related pathways.

### Effect of stanozolol on Smad2/3 phosphorylation and forkhead box O3a nuclear translocation in porcine muscle satellite cells

The phosphorylation level of Smad2/3 was decreased in PMSCs treated with 1 μM STZ compared with that of the control group at 24 h (p<0.01) (Figure 5A). Additionally, treatment of cells with 1 μM STZ significantly reduced FoxO3a fluorescence intensity and the FoxO3a-positive area at both 1 and 24 h relative to the control group (p<0.001) (Figure 5B). Collectively, these findings demonstrated that STZ attenuates atrophy-associated signaling pathways, including Smad2/3 and FoxO3a, suggesting that STZ promotes myogenic differentiation of PMSCs through a dual mechanism involving the activation of hypertrophy-related signaling and the concurrent suppression of atrophy-related pathways.

## DISCUSSION

The skeletal muscle is the principal component of livestock-derived meat and plays a major role in determining its structural characteristics and overall quality, including texture, tenderness, flavor, and processing properties [16,17]. Accordingly, the formation of muscle tissue through cell culture that mimics the structural and biochemical properties of conventional meat is essential for cultured meat production [18]. However, MSCs progressively lose their differentiation capacity during *in vitro* expansion, resulting in a decline in myotube formation efficiency over time. This limitation may hinder cultured meat production by reducing the ability to replicate the muscle fiber structure and protein composition of conventional meat. Therefore, the efficient myogenic differentiation of MSCs is critical for the generation of mature myofibers that determine the structural properties of the final cultured meat product [19]. Previous studies have explored various bioactive molecules and medium supplements, including INS, FGF-2, IGF-1, and naringenin, to promote stem cell differentiation into mature muscle fibers [20,21]. Nevertheless, current culture media formulations remain insufficient to support stable long-term culture and optimal differentiation of myogenic cells [21]. This suggests that more effective culture medium strategies are required to enhance myogenic differentiation and improve cultured meat production. In this context, STZ-induced enhancement of myogenic differentiation of PMSCs may contribute to improved production of cultured meat. Furthermore, STZ can be produced at low cost through a simple and efficient two-step synthetic process with a high overall yield [22]. Therefore, our study focused on the potential of STZ as a medium supplement to enhance myogenic differentiation and hypertrophic maturation of PMSCs. Our findings suggest that STZ supplementation in culture media is a promising and cost-effective strategy for enhancing cultured meat production by promoting myogenic differentiation.

In the present study, STZ did not induce cytotoxicity or cellular morphological changes in PMSCs at concentrations up to 1 μM, suggesting that STZ is safe *in vitro* within this concentration range. However, STZ did not increase the proliferation of PMSCs. Although androgenic stimulation has been reported to increase satellite cell proliferation, these effects are inconsistent across different experimental systems [8]. Furthermore, STZ has been reported to promote the proliferation of Michigan Cancer Foundation-7 breast cancer cells [10]; however, its effects on MSC proliferation remain unknown. Based on our data and previous reports, we conclude that the anabolic effects of STZ are primarily mediated by the promotion of myogenic differentiation rather than by PMSC proliferation. We characterized these promyogenic effects by evaluating MRF expression and analyzing signaling pathways associated with muscle hypertrophy and atrophy.

Myotubes are multinucleated structures formed by the fusion of myoblasts and serve as the foundation for the development of mature muscle fibers. However, inefficient myofusion remains a major limitation in cultured meat production because a substantial proportion of myogenic cells fail to fuse into myotubes, thereby limiting muscle maturation [23]. Therefore, efficient myotube formation is important for successful myogenic differentiation and contributes to the development of muscle-like structures. MyHC is a contractile protein associated with muscle development and differentiation and is expressed in developing muscle fibers [23,24]. Increased MyHC-positive fluorescence is indicative of enhanced terminal myogenic differentiation and myotube formation [23]. In addition, the fusion index is used to assess the proportion of myoblasts that can fuse into multinucleated myotubes and reflects myogenic differentiation capacity. A higher fusion index indicates a greater fusion capacity and is associated with enhanced myotube maturation [25]. Our data showed that STZ treatment increased MyHC fluorescence intensity and promoted multinucleated myotube formation in a concentration-dependent manner. Furthermore, 1 μM STZ treatment increased both the MyHC-positive area and fusion index compared with those of the control group. Collectively, our data demonstrated that 1 μM STZ is the most effective concentration for enhancing terminal myogenic differentiation and myotube maturation.

The myogenic differentiation of MSCs is regulated by the sequential activation of MRFs, which induces the differentiation of MSCs into myoblasts. These myoblasts subsequently fuse to form multinucleated myotubes that mature into functional myofibers [26]. Key MRFs, including myogenic factor 5, myogenic factor 6, MyoD and MyoG, regulate muscle differentiation and development throughout myogenic process [27]. MyoD and MyoG are key myogenic transcription factors that, when induced by myogenic signaling, drive the transition to terminal differentiation. MyoD initiates the myogenic process, whereas MyoG promotes the subsequent maturation and fusion of cells. MyHC, a major contractile protein upregulated during terminal differentiation, serves as a definitive marker of multinucleated myotube formation and structural integrity [25]. In the present study, STZ enhanced the myogenic differentiation of PMSCs by increasing the MyoD, MyoG, and MyHC mRNA levels on days 1 and 7. In addition, STZ reduced MSTN mRNA levels on days 1 and 7. MSTN, a muscle-derived myokine, acts as a potent negative regulator of myogenesis that is highly expressed in skeletal muscles and restricts muscle development by inhibiting MSC differentiation [28]. Furthermore, STZ increased MyoD and MyoG protein expression on days 1 and 7 compared with that in the control group. These findings suggest that STZ promotes PMSC myogenic differentiation by upregulating expression of MRFs (MyoD, MyoG, and MyHC) and concurrently suppressing that of MSTN. Notably, PMSCs treated with 1 μM STZ formed significantly larger and more robust myotubes by day 7, indicating that 1 μM STZ is an effective concentration for inducing terminal myogenic maturation. Collectively, our data indicate that STZ promotes the myogenic differentiation of PMSCs by upregulating expression of MRFs during both the early and late stages of myogenesis, whereas suppressing atrophy-associated signaling.

We evaluated the phosphorylation status of the PI3K/AKT/mTOR pathway 1 and 24 h after STZ treatment to determine whether the response was induced early and maintained over time. Our data indicated that STZ treatment significantly increased the phosphorylation of PI3K, AKT, and mTOR at 1 h, and this activation was sustained for 24 h compared with that observed in the control group. Skeletal muscle homeostasis is maintained by a balance between anabolic protein synthesis and catabolic protein degradation, which is governed by intracellular signaling pathways [29]. Protein phosphorylation is an early signaling response associated with skeletal muscle homeostasis. The key regulators of muscle growth and atrophy, including PI3K/AKT/mTOR, FoxO3a, and Smad2/3, are regulated by phosphorylation-dependent mechanisms [30]. Hypertrophic growth in skeletal muscles is achieved by increasing muscle fiber volume through enhanced protein synthesis [31,32]. This hypertrophic response is coordinated by the PI3K/AKT/mTOR signaling pathway, in which PI3K activation triggers AKT-mediated mTOR activation [31]. AKT maintains muscle mass by activating mTOR and inhibiting forkhead box O (FoxO) transcription factors, thereby suppressing protein degradation [33]. mTOR serves as a central regulator of muscle hypertrophy by integrating anabolic signals essential for myofiber expansion and muscle growth [34]. The PI3K/AKT/mTOR pathway responds rapidly to stimulation and sustains downstream signaling for several hours. In human tissues, phosphorylation changes in the PI3K/AKT/mTOR signaling pathway have been observed within minutes to hours and have been shown to persist for up to 24 h [35]. The PI3K/AKT/mTOR pathway is also activated during the myogenic differentiation of porcine myoblasts, although the time course of its phosphorylation remains unclear [36,37]. Collectively, our data suggest that STZ treatment promotes myogenic differentiation by rapidly activating the PI3K/AKT/mTOR signaling pathway and sustaining its activation at later time points. Notably, androgenic responses in porcine satellite cells may be context-dependent, as testosterone has been reported to upregulate AR expression but reduce differentiation in porcine myogenic satellite cells [38]. Therefore, the promyogenic effect of STZ in PMSCs under cultured meat-relevant differentiation conditions cannot be assumed solely from the general anabolic properties of AASs. Although the present study showed that STZ enhanced myogenic differentiation and activated PI3K/AKT/mTOR signaling in PMSCs, the upstream receptor-mediated mechanism was not directly confirmed. STZ has been reported to act as a potent AR activator despite its relatively low AR-binding affinity, and previous studies have shown that androgen/AR signaling can regulate myogenic differentiation and interact with mTOR-related hypertrophic signaling [7–9,39]. Therefore, the STZ-induced activation of PI3K/AKT/mTOR signaling observed in our study may be partly associated with AR-related signaling. Further study is necessary to determine whether AR activation is necessary for STZ-induced myogenic differentiation and myotube formation in PMSCs.

Muscle atrophy is driven by activation of the FoxO transcription factors and MSTN-induced Smad2/3 signaling, which upregulate atrophy-related ubiquitin ligases, accelerate proteolysis, and ultimately lead to muscle loss [40]. Phosphorylated Smad2/3 forms a complex with Smad4 and translocates to the nucleus, where it cooperates with FoxO transcription factors to induce the expression of genes associated with muscle wasting [41,42]. Smad2/3 and FOXO proteins can act as downstream effectors of PI3K signaling, and their phosphorylation status can be modulated under conditions that alter PI3K activity [42]. Therefore, we evaluated Smad2/3 phosphorylation and FoxO3a expression 1 and 24 h after STZ treatment to assess the early response and the extent of the response at later time points. Our data showed that STZ treatment decreased Smad2/3 phosphorylation at both 1 and 24 h and reduced FoxO3a fluorescence intensity and FoxO3a-positive area relative to the control group. These data indicate that STZ treatment promotes myogenic differentiation by suppressing atrophy-associated signaling molecules, including Smad2/3 and FoxO3a.

Although STZ effectively promoted myogenic differentiation and myotube formation in PMSCs, its application to cultured meat production should be considered carefully because STZ belongs to the AAS family [43]. From a regulatory and food safety perspective, when steroidal compounds are considered as culture medium supplements, their residual levels, metabolic fate, and potential exposure in the final edible product should be carefully evaluated [43,44]. In addition, consumer acceptance of cultured meat can be influenced by perceived safety, naturalness, and food-related risk perception, highlighting the need for assessment and communication regarding the use of steroid-related compounds during production [45]. Therefore, further research is necessary to investigate STZ residue profiles, dose-minimization strategies, and whether STZ or its metabolites remain in harvested cells after medium replacement and washing steps before practical application in cultured meat systems.

Collectively, our findings demonstrate that STZ promotes myogenic differentiation and maturation of PMSCs through a dual mechanism involving activation of the PI3K/AKT/mTOR hypertrophic pathway and suppression of atrophy-associated signaling, including Smad2/3 and FoxO3a. These data suggest that STZ is a promising medium supplement for improving the efficiency of myotube formation and enhancing cultured meat production.

## CONCLUSION

In this study, we evaluated the potential of STZ as a medium additive to improve the myogenic differentiation and maturation of PMSCs for cultured meat production. STZ showed no significant cytotoxicity in PMSCs within the tested concentration range and did not affect PMSC proliferation. However, STZ effectively promoted myogenic differentiation at both the early (day 1) and late (day 7) stages by increasing the expression of key myogenic markers such as MyoD, MyoG, and MyHC, whereas suppressing MSTN expression, thereby enhancing multinucleated myotube formation. Notably, 1 μM STZ formed the greatest number of multinucleated myotubes. Furthermore, STZ activated hypertrophy-related PI3K/AKT/mTOR signaling as early as 1 h and was maintained for up to 24 h, while concurrently attenuating atrophy-associated signaling by reducing Smad2/3 phosphorylation and FoxO3a activity at both 1 and 24 h. Collectively, these findings suggest that STZ is a promising medium supplement for enhancing myotube formation in cultured meat. However, the receptor-mediated mechanisms and the performance of 3D systems remain unclear. Further research is necessary to validate these effects across additional livestock species and scalable systems, while conducting rigorous safety and sensory assessments. Such studies are essential to confirm the practical viability of STZ as a cost-effective strategy for large-scale high-quality cultured meat production.

## Figures and Tables

**Figure 1:**
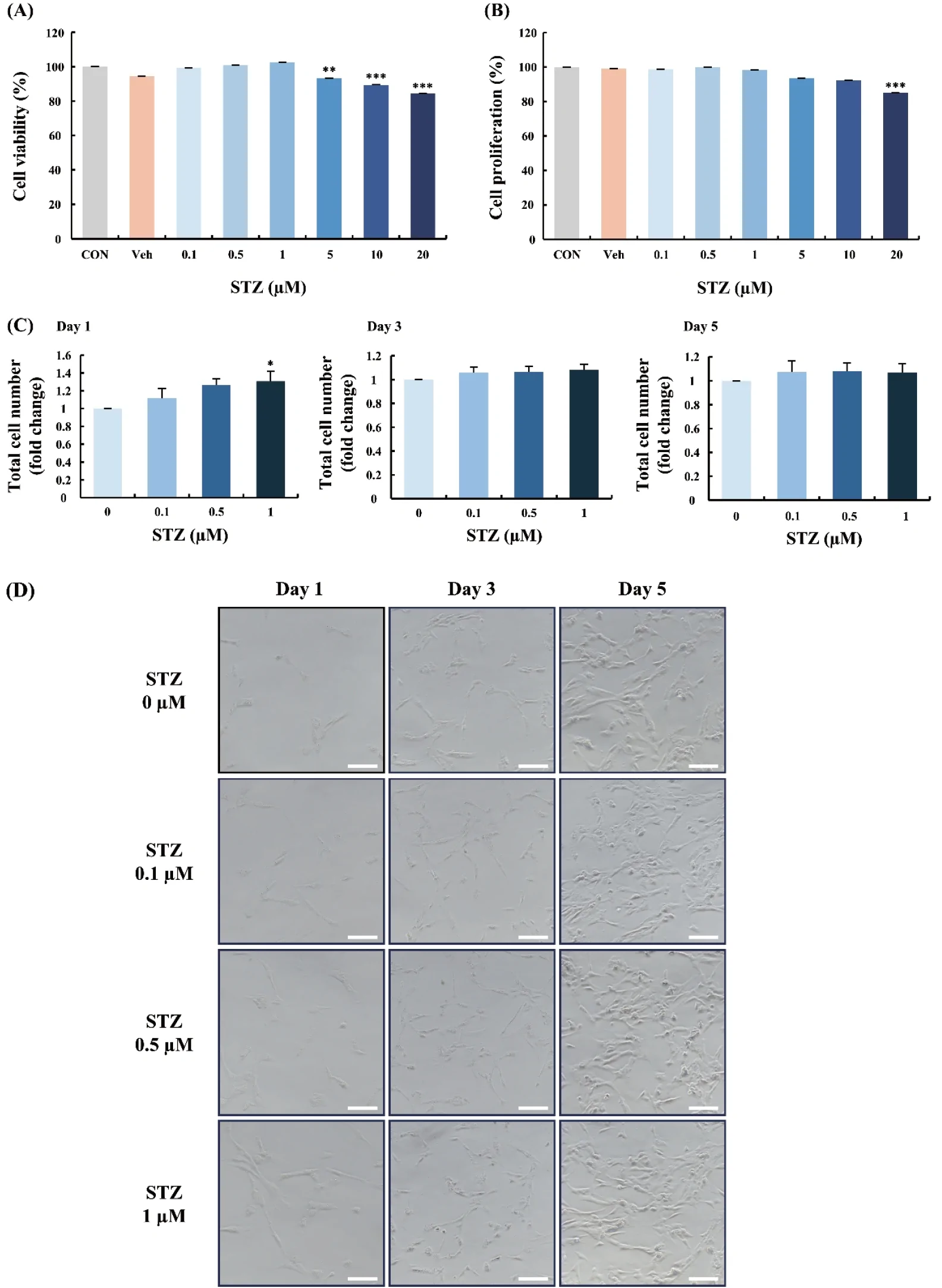
Effects of stanozolol (STZ) on cytotoxicity and proliferation in porcine muscle satellite cells (PMSCs). PMSCs were treated with STZ at concentrations ranging from 0.1 to 20 μM. STZ was dissolved in dimethyl sulfoxide (DMSO), and vehicle controls (Veh) contained 0.2% DMSO as indicated. (A) Cell viability was assessed using MTT assay after 24 h. (B) Cell proliferation was evaluated using CCK assay after 48 h. In 1 μg/mL insulin-supplemented conditions, PMSCs were treated with STZ (0.1, 0.5, and 1 μM); (C) total cell number and (D) morphological changes were examined on days 1, 3, and 5. Images were captured at 100× magnification (scale bar indicates 100 μm). Data are represented as mean±standard error (n = 3). * p<0.05, ** p<0.01, and *** p<0.001 denote statistical significance vs. control. MTT, 3-(4,5-Dimethylthiazol-2-yl)-2,5-diphenyl-2H-tetrazolium bromide; CCK, cholecystokinin.

**Figure 2:**
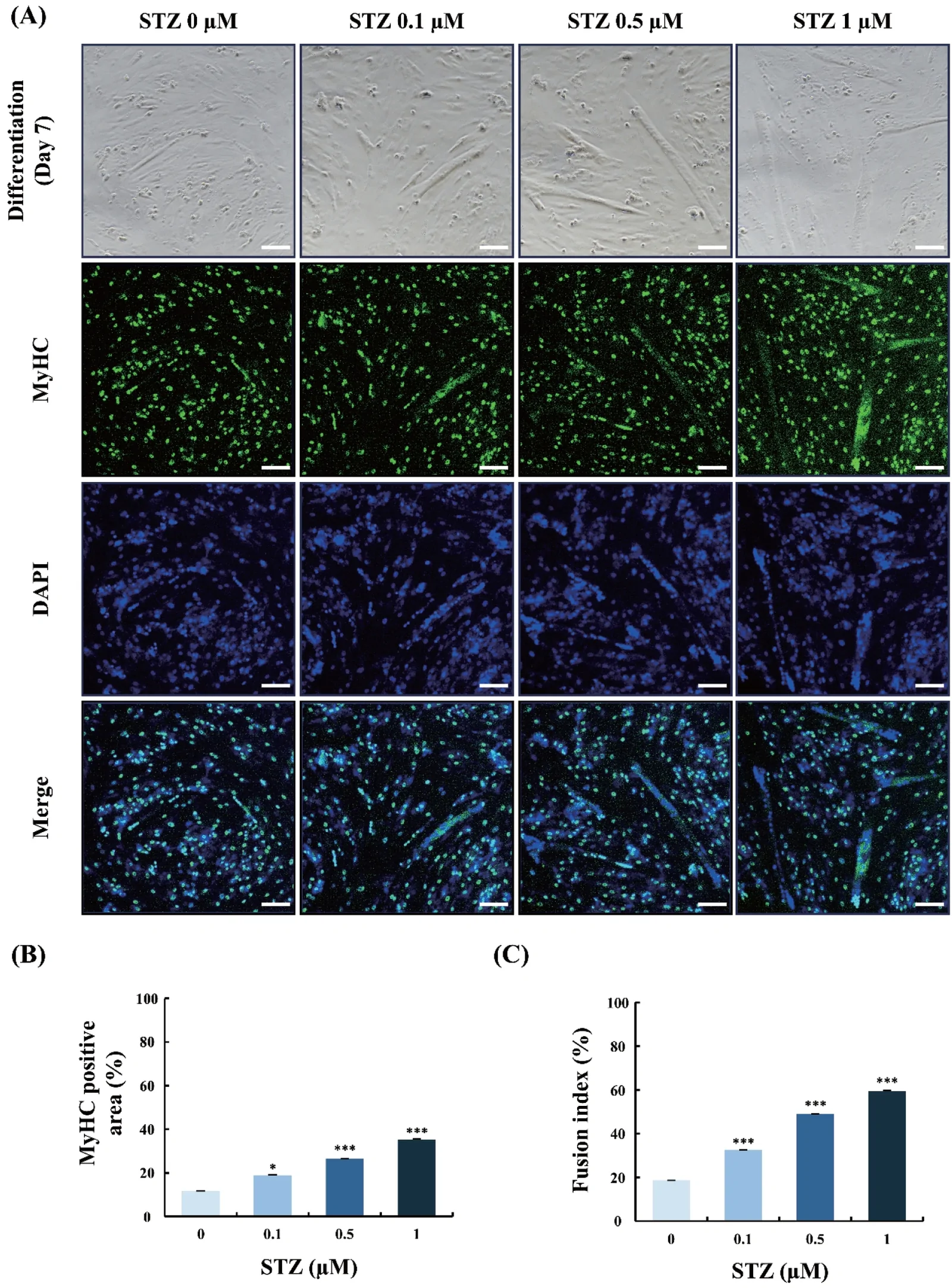
Assessment of the effects of STZ on myotube formation in PMSCs. PMSCs were treated with 1 μg/mL insulin and STZ (0.1, 0.5, and 1 μM) for 7 days. (A) Representative fluorescence images of MyHC were captured along with corresponding bright-field and DAPI-stained images. MyHC and DAPI were visualized with green and blue fluorescence, respectively. (B) Quantification of MyHC-positive area expressed as a percentage of the total field area. (C) Fusion index calculated as the percentage of nuclei within myotubes relative to total nuclei. Quantification was performed using ImageJ software. Images were captured at 100× magnification (scale bar indicates 50 μm). Data are represented as mean±standard error (n = 3). * p<0.05 and *** p<0.001 denote statistical significance vs. control. STZ, stanozolol; PMSCs, porcine muscle satellite cells; MyHC, myosin heavy chain; DAPI, 4′,6-diamidino-2-phenylindole dihydrochloride.

**Figure 3:**
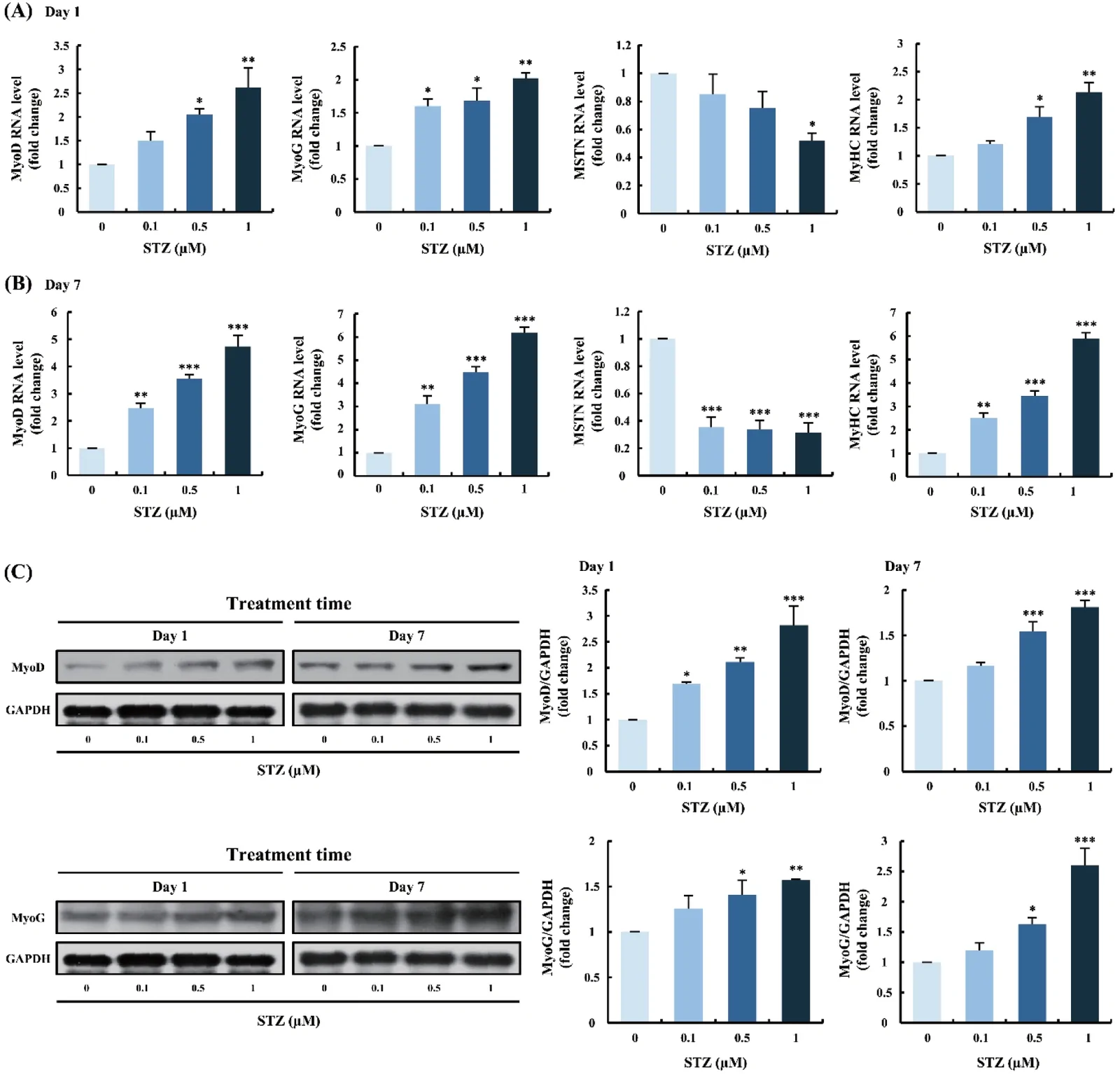
Evaluation of the effects of STZ on myogenic regulatory factors in PMSCs. PMSCs were treated with 1 μg/mL insulin and STZ (0.1, 0.5, and 1 μM), and samples were collected on days 1 and 7. mRNA expression levels of MyoD, MyoG, MSTN, and MyHC were measured by RT-qPCR at days (A) 1 and (B) 7. (C) Protein expression levels of MyoD and MyoG were analyzed by western blot on days 1 and 7. GAPDH was used as the housekeeping protein. Data are represented as mean±standard error (n = 3). * p<0.05, ** p<0.01, and *** p<0.001 denote statistical significance vs. control. MyoD, myogenic differentiation 1; STZ, stanozolol; MyoG, myogenin; MSTN, myostatin; MyHC, myosin heavy chain; GAPDH, glyceraldehyde 3-phosphate dehydrogenase; PMSCs, porcine muscle satellite cells; RT-qPCR, real-time polymerase chain reaction.

**Figure 4:**
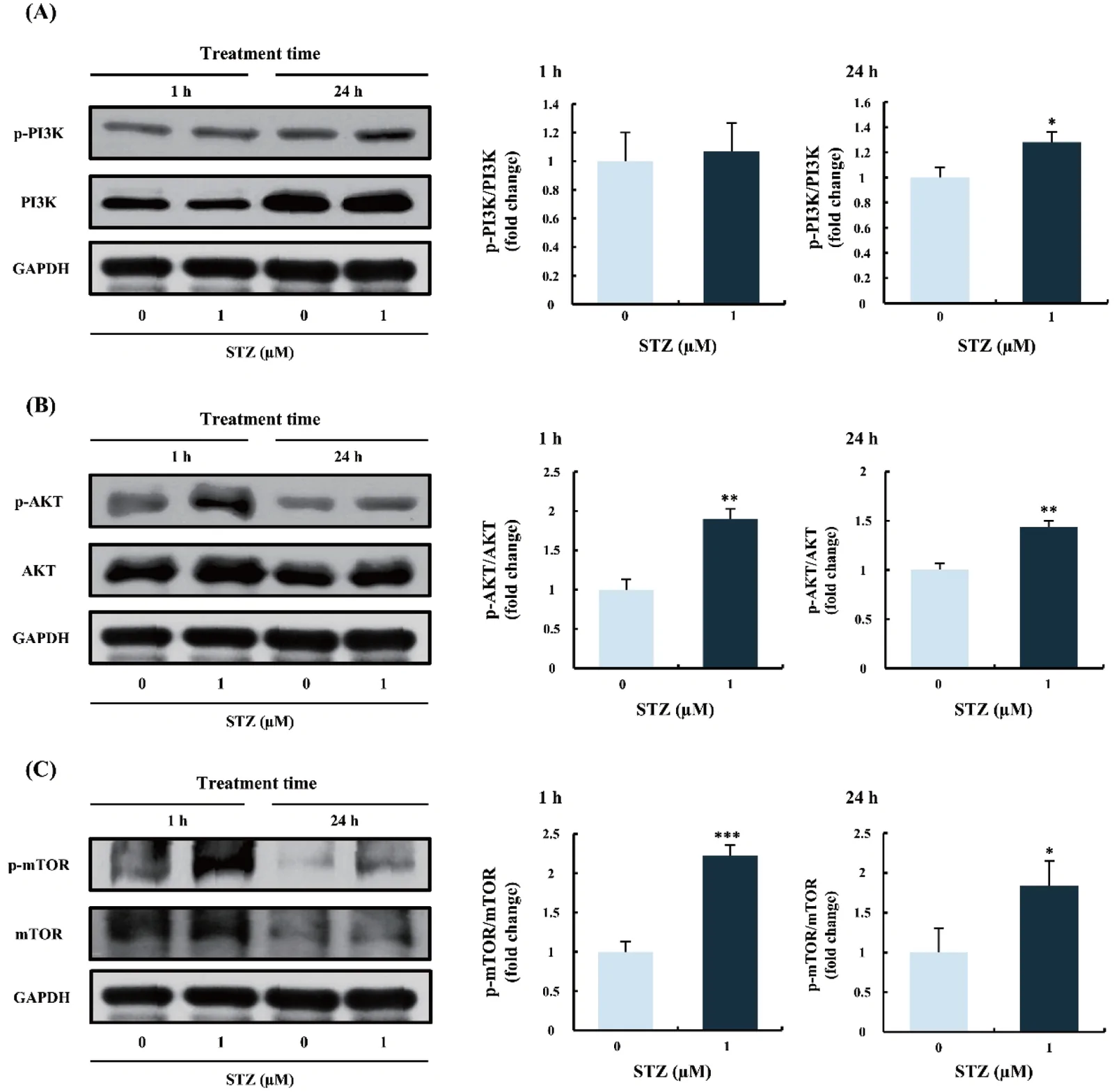
Western blot of muscle hypertrophy signaling in PMSCs treated with STZ. PMSCs were treated with 1 μg/mL insulin and 1 μM STZ for 1 h or 24 h. The expression levels of phosphorylated (A) PI3K, (B) AKT, and (C) mTOR were analyzed by western blot. Phosphorylation levels were normalized to the corresponding total protein levels. GAPDH was used as a loading control. Data are represented as mean±standard error (n = 3). * p<0.05, ** p<0.01, and *** p<0.001 denote statistical significance vs. control. PI3K, phosphatidylinositol 3-kinase; GAPDH, glyceraldehyde 3-phosphate dehydrogenase; STZ, stanozolol; AKT, serine/threonine kinase; mTOR, mammalian target of rapamycin; PMSCs, porcine muscle satellite cells.

**Figure 5:**
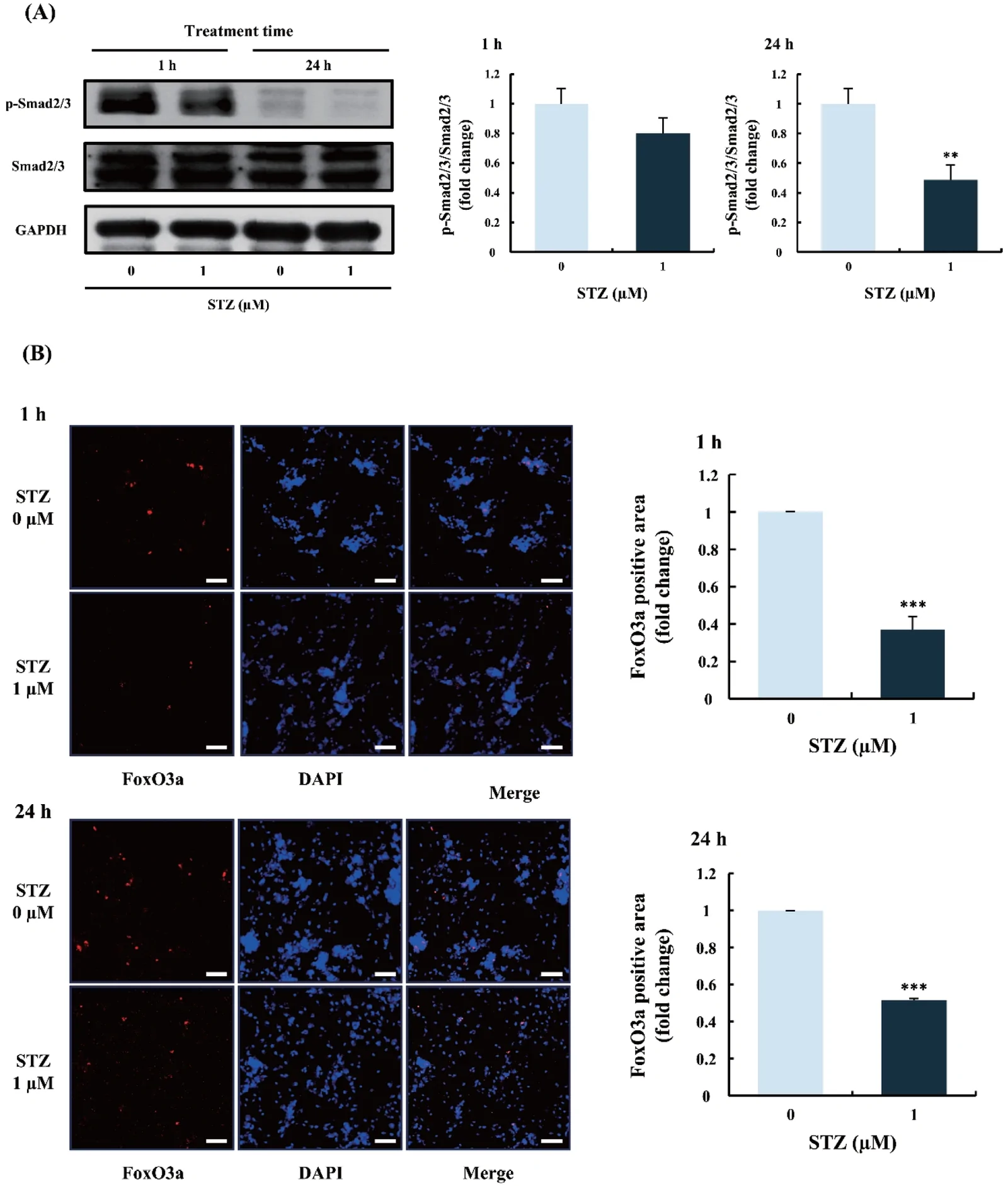
Muscle atrophy signaling in PMSCs treated with STZ. PMSCs were treated with 1 μg/mL insulin and 1 μM STZ for 1 h or 24 h. (A) The expression levels of phosphorylated Smad2/3 were analyzed by western blot. (B) Representative fluorescence images of FoxO3a were captured along with corresponding DAPI-stained images. FoxO3a and DAPI were visualized with red and blue fluorescence, respectively. Representative images are shown on the left, and quantification of the FoxO3a-positive area is shown on the right. Phosphorylation levels were normalized to the corresponding total protein levels. GAPDH was used as a loading control. Quantification was performed using ImageJ software. Images were captured at 100× magnification (scale bar indicates 100 μm). Data are represented as mean±standard error (n = 3). ** p<0.01, and *** p<0.001 denote statistical significance vs. control. GAPDH, glyceraldehyde 3-phosphate dehydrogenase; STZ, stanozolol; FoxO3a, forkhead box O3a; DAPI, 4′,6-diamidino-2-phenylindole dihydrochloride; PMSCs, porcine muscle satellite cells.

## Data Availability

Upon reasonable request, the datasets of this study can be available from the corresponding author.
